# Caries Experience and Oral Health-Related Habits in Blind and Low-Vision Individuals in Croatia

**DOI:** 10.3390/jcm14155576

**Published:** 2025-08-07

**Authors:** Jelena Dumančić, Marijana Radić Vuleta, Božana Lončar Brzak, Ivana Savić Pavičin, Tara Kurpez, Neda Striber, Ivana Čuković-Bagić

**Affiliations:** 1 Department of Dental Anthropology, School of Dental Medicine, University of Zagreb, 10000 Zagreb, Croatia; savic@sfzg.unizg.hr; 2 Department of Dental Medicine, University Hospital Centre Zagreb, 10000 Zagreb, Croatia; marijana.radic@hzjz.hr (M.R.V.); cukovic-bagic@sfzg.unizg.hr (I.Č.-B.); 3 Croatian Institute of Public Health, 10000 Zagreb, Croatia; 4 Department of Oral Medicine, School of Dental Medicine, University of Zagreb, 10000 Zagreb, Croatia; loncar@sfzg.unizg.hr; 5 Stichting Bijzondere Tandheelkunde (Center for Special Care Dentistry), 1081 LA Amsterdam, The Netherlands; t.kurpez@sbt.nl; 6 Children’s Hospital Zagreb, 10000 Zagreb, Croatia; neda.striber@kdb.hr; 7 Department of Paediatric and Preventive Dentistry, School of Dental Medicine, University of Zagreb, 10000 Zagreb, Croatia

**Keywords:** blindness, visual impairment, dental caries, oral health, public health, Croatia, disability

## Abstract

**Objectives**: The aim of the study was to investigate caries experience in correlation with self-reported oral health-related habits in a sample of blind and low-vision individuals in Croatia. **Methods**: The study is a part of the research in the “Project for Oral Health Promotion in Blind and Visually Impaired Persons” conducted at the Zagreb University School of Dental Medicine from 2014 to 2018. The final sample consisted of 85 adults: 42 females and 43 males; 50 blind and 35 low-vision individuals, age range 18–98. The assessment included dental examination and a questionnaire. **Results**: The median DMFT (Decayed, Missing, and Filled Teeth) index score was 17.0 (IQR = 12.5–22.0), with no significant difference between sexes or between blind and low-vision individuals. The occurrence of untreated caries was low (median D-component = 1.0), while the median F-component was 6.0. There was a significant increase in M-component and DMFT in older age groups. The number of untreated caries (D-component) was significantly correlated with consummation of soft drinks and smoking. Total DMFT did not correlate with frequency of tooth brushing, time since last dental visit, smoking, or level of education. **Conclusions**: This study revealed high caries experience among blind and visually impaired individuals that did not correlate with factors that normally influence oral health. Similar results were found in the control group, reflecting a 30-year post-war period without organized preventive care. The low number of decayed teeth reflects the availability of public dental care in Croatia; however, preventive care should be provided for both this vulnerable group and the general population.

## 1. Introduction

Oral health is an important component of overall well-being, significantly influencing daily activities and quality of life. Although oral diseases are mostly preventable, some populations are at higher risk of developing disease, and many of the risk factors are largely beyond individuals’ direct control [1,2]. People with disabilities are one of the vulnerable groups in health and social care that require special support [3].

Globally, the World Health Organization (WHO) estimates that there are at least 2.2 billion people with vision impairment or blindness, affecting individuals of all ages [4]. According to the WHO definition, blindness is specified as visual acuity less than 20/400, with the best possible correction, or a remaining visual field of 10° or less in the better-seeing eye. Low vision is visual acuity of 20/70 to 20/400 (inclusive), with the best possible correction [5]. It is estimated that there are over 30 million blind and visually impaired people in Europe. On average, 1 in 30 Europeans experiences vision loss [6]. According to the Croatian Register of Persons with Disabilities, 21,728 people are visually impaired, while 7407 people experienced vision loss [7].

Visual impairment affects various aspects of life, including sensory perception, mobility, social interactions, and access to written information. Moreover, vision loss can significantly increase the risk of falls, injuries, and decline in mental health, cognition, and educational achievement [8,9]. Visually impaired people may be at a higher risk of developing oral diseases, including caries and periodontal disease, due to various limitations in activities and perception. These include a lack of hand–eye coordination and consequent difficulties in maintaining effective oral hygiene, inability to detect dental plaque and early oral disease, lack of aesthetic perception of teeth and mouth, and mobility problems and the need for assistance, which affect visiting dentists.

An important factor in the perception of oral health among people with disabilities is their subjective experience, which manifests in numerous challenges in receiving effective dental care. Barriers such as lack of transportation, lack of accessible dental clinics, and deficiencies in the healthcare system that inadequately address the needs of this vulnerable population often result in delayed or completely neglected dental care [10,11,12,13,14].

Over the last 50 years, many documents have been adopted to enable equal opportunities for people with disabilities, and in December 2006 the United Nations General Assembly adopted the Convention on the Rights of Persons with Disabilities, which was signed and ratified by most of the countries worldwide [15]. One of the human rights protected by the Convention is the right to healthcare of the same scope, quality, and standard as provided to other citizens. In relation to dental care, this has not yet been achieved, as patients with disabilities are mostly treated in special clinics or hospitals, with long waiting lists. It is estimated that 90% of people with disabilities should be able to access dental treatment in primary care settings, while only the most complex cases need specialist or hospital care. The prerequisite is to include education in special care in undergraduate dental curricula to equip future dentists with the knowledge, skills, and attitudes necessary to meet the oral health needs of vulnerable groups [16,17,18].

Despite the high prevalence of visual impairment, there is little available information regarding the oral health of visually impaired adults. Most research indicates poorer oral health, caused by mobility challenges, the need for assistance, and the lack of aesthetic perception of teeth and mouth [19,20,21,22,23,24,25]. Several studies have shown insufficient oral health knowledge in visually impaired people, who are more likely to visit dentists only when experiencing pain [22,26,27].

In accordance with the WHO Global strategy and action plan on oral health 2023–2030 and the United Nations Convention, it is necessary to advance research on people with disabilities to identify the limitations and their consequences in receiving dental health care as well as in maintaining good oral health [15,28,29]. By understanding the unique challenges they face, targeted interventions can be developed to improve oral health and quality of life.

At the Zagreb University School of Dental Medicine, an oral health promotion program titled “Project for oral health promotion in blind and visually impaired persons” was designed and conducted from 2014 to 2018 [30,31]. The main objectives were (1) to evaluate and improve oral health in blind and low-vision individuals, (2) to develop sensitivity toward people with disabilities in dental students and professionals, and (3) to design the content of undergraduate training in special care dentistry. A secondary objective was to assess oral health in blind and low-vision individuals. The Project offered dental examinations, prophylaxis, preventive procedures, and oral hygiene instructions in an individual approach to participants. Education on oral health was also organized in the form of public lectures and panel discussions for adults and workshops and playschool for children. Student volunteers joined the Project team and participated in clinical work, interviews, and acted as sighted guides.

In the present research, we assess caries experience among blind and low-vision individuals, participants in the Project for Oral Health Promotion in Blind and Visually Impaired Persons (henceforth referred to as the Project), and evaluate its possible relationship with age, sex, education, and oral health-related habits, in comparison to a control group of individuals with normal vision and no intellectual, psychological, or physical disability.

## 2. Materials and Methods

The present research is an observational cross-sectional study conducted on 85 non-institutionalized visually impaired people, mostly from the capital of Croatia, Zagreb—participants of the Project.

Call for blind and visually impaired people was distributed in a form of invitation letter sent by regular mail and email, and through announcements on the Web page of the Croatian Blind Union and the Zagreb Association of the Blind in 2014, 2015, and 2017. The information service of the City of Zagreb distributed the news about the start of the Project to local media, radio, and news portals, and presentation of the Project was organized at the Zagreb Association of the Blind.

Inclusion criteria were blindness or low vision. Exclusion criteria included conjoint intellectual, psychological, or physical disability. Eighty-seven adults voluntarily applied to enrol in the Project. With the exclusion of two individuals (non-cooperative and dependent), the final sample consisted of 85 self-selected participants (Table 1).

After taking medical and dental history, Project participants received oral examinations (dental, periodontal, mucosal, and oral hygiene status), prophylaxis, preventive procedures, and oral hygiene instructions in an individual approach.

Oral health questionnaire

The WHO Oral Health Questionnaire for Adults was filled in the form of an interview, with the assistance of dental students [32]. The questionnaire included questions regarding socio-demographic characteristics, sex, age, place of residence, education, oral hygiene habits, frequency and reason for dental visits, frequency of consumption of sugary foods and drinks, and tobacco and alcohol consumption.

Clinical dental examination

Dental examinations were carried out at the University School of Dental Medicine Clinic using a dental probe and plain mouth mirror. Clinical diagnostic criteria for dental caries included lesions with cavitation, in accordance with WHO criteria [32]. Caries experience was recorded as the number of decayed (D), missing (M), and filled (F) teeth (DMFT index). Calculations were based on 32 teeth. Radiographs were taken only if clinically indicated and not for the purpose of this study. All participants were informed about their oral health status and received recommendations for further treatment and findings in written form. Dental status was assessed by three calibrated clinical examiners (J.D., B.L.B. and I.S.P.). In each session, two examiners worked together, one of whom was the Project leader (J.D.), who supervised and validated all cases.

Control group

Control group recruitment was delayed due to the COVID-19 pandemic and was carried out during 2023 and 2024. Regular patients from the Department of Family Dentistry, Dental Clinic, University Hospital Centre Zagreb, and the private clinic Dentclarus were invited to participate as a control group, matched with study group by age, sex, and education level. The control group comprised 85 individuals with no visual impairment, who consented to a clinical examination and questionnaire. The same exclusion criteria were applied as for the Project participants. All individuals received prophylaxis, preventive procedures, and oral hygiene instructions, same as the study group. Examinations were performed by the Project leader (J.D.) and by a young investigator (M.R.V.), who was previously trained by the Project leader.

The examiners were calibrated prior to and midway through data collection, and on both occasions the inter- and intra-examiner intraclass correlation coefficients (ICC) ranged between 0.95 and 0.98, indicating a very high level of agreement.

Ethical clearance

The research was conducted in accordance with the guidelines of the Declaration of Helsinki and approved by the Ethics Committee of the School of Dental Medicine University of Zagreb in 2014 for the Project and 2019 for the inclusion of the control group. Informed consent was obtained from all participants.

Statistical analysis

Statistical analysis was performed using IBM (Armonk, NY, USA) SPSS Statistics for Windows, version 29.0.1., with a *p*-value lower than 0.05 considered statistically significant. The Kolmogorov–Smirnov test was used to assess the normality of data distribution. The Mann–Whitney U test was used to evaluate change in the DMFT index in relation to diagnosis (blind or low-vision individuals) and sex, while the Kruskal–Wallis H test was used to test relation with age, place of residence, and education level. Correlation between DMFT and oral health-related habits was tested using the Spearman correlation coefficient. The Fisher–Freeman–Halton’s exact *p* test was used to compare the study group with the control group for place of residence, clinical parameters, and oral health-related habits.

## 3. Results

Demographics and characteristics

Demographic data are listed in Table 2. There was almost equal distribution of the sample by sex. The age range was 18–98 years (median = 54.0, IQR = 33.5–66.0). The distribution by age groups shows that the largest group consisted of middle-aged individuals between 45 and 64 years (29%), followed by the 18–34 and 65–74 age groups, comprising approximately 25% each. The place of residence for the majority was urban (82%), while for the control group, the distribution by place of residence was significantly different, with 66% residing in urban, 28% in peri-urban, and 6% in rural areas (*p* = 0.025, Fisher–Freeman–Halton exact *p*).

The majority of participants (52%) completed secondary school, while 27% had college or university degrees.

### 3.1. Caries Experience in Study and Control Groups

The DMFT scores and their components for the study and control groups are presented in Table 3. The median DMFT value for the study group was 17.0 (IQR = 12.5–22.0). The occurrence of untreated caries was very low, with a median D component 1 (IQR = 0–3.0). For DMFT and F, slightly lower values were found in the control group; however, there was no significant difference between the groups.

### 3.2. Study Group Comparison in DMFT by Categories of Visual Impairment, Sex, Age, Place of Residence, and Education

Table 4 shows the DMFT scores and their components in study individuals, distributed by categories of visual impairment, sex, age, place of residence, and education. The median DMFT did not differ significantly between the two groups according to visual impairment (blind vs. low vision), nor between sexes, although males showed slightly higher M and F median values.

DMFT and M scores increased with age, and the difference between age groups was highly significant (*p* < 0.001, Kruskal–Wallis test). D, M, and DMFT were higher among individuals from rural settings, but there was no significant difference.

When study individuals were compared according to level of education, no significant difference in DMFT was found.

### 3.3. Oral Health-Related Habits—Questionnaire Results

**Figure 1:**
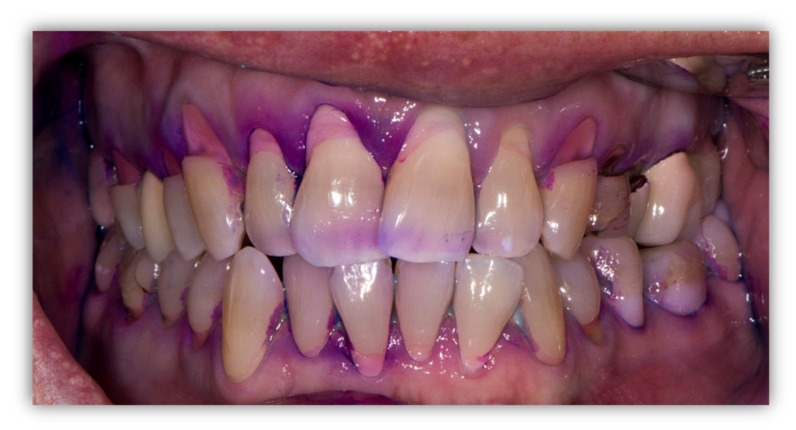
Intraoral photo of the dentition of a 45-year-old blind male participant in the Project: frontal view after plaque revelator application. Wedge-shaped tooth defects and gingival recessions caused by excessive toothbrushing are visible. No plaque is revealed on labial and buccal surfaces; however, newly deposited and mature plaque is visible interdentally, coloured in violet and blue, respectively, indicating lack of interdental cleaning. Although there is evidence of dental work, many extremely deep wedge-shaped defects were left untreated.

Table 5 presents a comparison between the study and control groups in the experience of toothache, frequency and reasons for dental visits, and oral hygiene habits. More than half of individuals with visual impairment (58%) reported experiencing toothache/discomfort in the last year, which was less frequent than in control individuals (non-significant difference). In both the study and control groups, the reported frequency of toothbrushing twice per day and more was 73%. There was a significantly higher awareness and greater use of fluoridated toothpaste reported in the blind and low-vision individuals (*p* < 0.001). Dental floss use was reported by 28% of individuals with visual impairment and 39% in the control group; however, no significant difference was found. Figure 1 presents the dental status of a 45-year-old blind male participant in the Project, who reported brushing his teeth two or more times a day but did not use dental floss. There are many wedge-shaped defects of teeth and gingival recessions due to excessive toothbrushing, so plaque-revealing agent stained new and mature dental plaque located only interdentally. There is evidence of dental work, including fillings and crowns, but many extremely deep wedge-shaped defects were left untreated.

Forty-one percent of participants reported visiting the dentist within the last 6 months, and an additional 21% in the past year. The most common reasons for visits were pain/troubles with teeth or gums (38%) and dental treatment (38%). Only 18% went for a regular dental checkup. Similar results were reported in the control group.

Table 6 presents the consumption of sugary foods and drinks. The study group reported daily consumption of fruit (59%), biscuits/cakes (19%), jam/honey (19%), sweets/candies (12%), tea/coffee with sugar (11%), and other sugary items (<10%).

There were only 15 (18%; seven males and eight females) daily tobacco smokers, and one female smoking several times per month. About 38% reported no alcohol consumption during the past month, and a further 42% reported having one or two drinks per day (Table 7). No statistical difference was observed between the study and control groups regarding dietary habits, tobacco use, and alcohol consumption.

### 3.4. Correlation of DMFT with Oral Health-Related Habits

**Figure 2:**
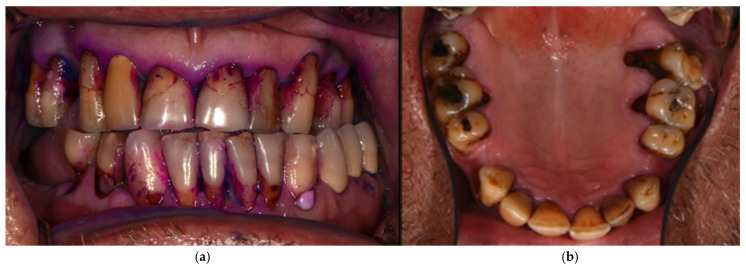
Poor oral/dental status of a 55-year-old smoker male participant in the Project: (**a**) frontal view of the dentition after plaque revelator application; (**b**) occlusal view of the upper dentition before application of plaque revelator (mirror image).

The correlation of DMFT with oral health-related habits in the study group was tested using Spearman’s correlation test (Table 8). Oral hygiene, evaluated by frequency of toothbrushing and use of fluoridated toothpaste, was not correlated with DMFT. The frequency of dental visits showed a highly significant correlation with the F-component, suggesting more filled teeth in individuals with more frequent dental visits (*p* = 0.000). The reasons for the last dental visit showed a significant negative correlation with the D and M components, suggesting more decayed and extracted teeth in individuals who visited the dentist because of pain caused by teeth/gums or dental treatment (*p* < 0.05). Results regarding the correlation between DMFT and dietary habits showed a highly significant correlation between decayed teeth (D-component) and the consumption of soft drinks and lemonade (*p* = 0.000). The number of decayed teeth was also in significant correlation with tobacco use (*p* = 0.014). Figure 2 presents the dental status of a male with low vision who was a smoker.

## 4. Discussion

Dental caries and its repercussions remain an important public health challenge in various vulnerable populations, including visually impaired people. We assessed caries experience and its possible relationship with socio-demographic characteristics and oral health-related habits in 85 non-institutionalized visually impaired individuals, mainly from Zagreb, who were participants in the Project for oral health promotion in blind and visually impaired persons. Analysis was conducted in comparison with a control group of individuals without disabilities, matched by sex, age, and level of education. This is the first study on dental health in visually impaired persons within the Croatian population, and to our knowledge, there are only several reports worldwide [20,23,33,34].

The present research resulted from a preventive program, the primary goals of which were related to the benefit and promotion of oral health in a vulnerable population of visually impaired individuals. The sample included 50 blind and 35 low-vision self-selected participants. Eight individuals (9%) had congenital blindness, while the others had either progressive visual impairment that resulted in blindness later in life or low vision (Table 1). There was an even number of male and female participants, and a relatively even distribution of participants across three out of five age groups (18–34, 45–64, and 65–74 years). A moderately high level of education was observed among the participants: 52% with secondary school, and 27% with college or university degrees, which could influence their understanding of health information, or other health-related behaviours. This relatively large number of highly educated people may reflect the population of the capital city, where education is more accessible.

### 4.1. Caries Experience

Our results showed high caries experience in visually impaired persons (median DMFT = 17), which was the same in totally blind and low-vision individuals, but, contrary to our expectations, not significantly higher than in the control group (median DMFT = 16). As our sample included only eight individuals (9%) who were blind from birth and in whom we can expect the greatest impact of disability on oral health, this may explain the resulting comparative caries experience as in the control group.

Our findings are consistent with other reported studies. Watson et al. found a similar dental status in visually impaired adults and the general United Kingdom (UK) population; however, they did not provide a DMFT score [20]. Possibly no difference was found due to the same limitation as in our study: a sample of visually impaired patients of the largest UK ophthalmic hospital in London, who probably had better access to health services, was compared to the data of the general UK population.

The DMFT level found in our study was very close to Brazilian data from 2009, which showed a median DMFT value of 12 among people aged 20–34 and 17.5 among people aged 35–50 years [33]. Another study conducted in Brazil also found high mean DMFT in groups aged 22–49 (15.3) and 50–80 (21.7), which aligns with our results [34].

There was a significant increase in DMFT and M scores with age (*p* < 0.001) showing the cumulative caries effect over the lifetime, which is consistent with the aforementioned studies [33,34]. A higher DMFT score in the elderly is expected due to various factors that lead to tooth decay and tooth loss: poor oral hygiene, dietary habits, systemic diseases, and the natural ageing process [35].

### 4.2. Questionnaire Results on Oral Health-Related Habits

The questionnaire results revealed that visually impaired individuals have similar oral hygiene and dental visit habits as the control group. The majority (73%) report brushing twice or more times a day; however, 72% do not floss, compared to 61% in the control group, which is expected, as flossing demands eye–hand coordination and is acquired as a fine motor skill. Surprisingly, awareness and reported use of fluoridated toothpaste were significantly higher in the blind and low-vision individuals (*p* < 0.001) compared to the controls; most of the sighted individuals were unaware whether their toothpaste contained fluoride. These results indicate a general lack of knowledge about simple and available preventive measures and a need for broader oral hygiene education in the Croatian population. During the Project, a lack of facial mimic was observed in totally blind participants, which prevented effective toothbrushing, as lips were not lifted from the dental arches. Additionally, some participants manifested excessive brushing with consequent wedge-shaped tooth defects (Figure 1). Each participant was instructed on dental flossing and toothbrushing using the “hand-over-hand” technique, with the patient holding the floss/toothbrush and the dental clinician/student physically guiding the patient’s hand. For such practical instruction, the dentist needs to plan time and prepare oral healthcare products, and the patient needs to wash their hands before sitting in the dental chair. This takes preparation, assistance and time; however, without everyday instruction on oral hygiene and motivation of patients, we cannot truly speak about patient-centred healthcare.

Regarding the frequency of dental visits in the last six months, results suggest that individuals with normal vision are slightly more likely to visit the dentist regularly, but this difference is not statistically significant and may be due to other factors like accessibility or awareness of dental aesthetics. Since visually impaired people cannot perceive subtle aesthetic changes, such as gingival colour variations or change of tooth/filling colour, they probably rely more on sensations of comfort or discomfort. Overall reasons for dental visits are quite similar, indicating that both groups seek dental care when they experience pain or require treatment, rather than for preventive care.

Previous studies have reported different levels of oral hygiene habits in visually impaired individuals. Watson et al. found that visually impaired adults were more likely to brush their teeth two or more times a day compared to the reference group, and significantly more likely to use dental floss (49% compared to 25%) [20]. Lee et al. investigated oral health-related habits among visually impaired students aged 6–21 from two specialized schools in Hong Kong [36]. They reported 90% of visually impaired students brushed twice a day, and 21% performed regular interdental cleaning.

Watson et al. report that visually impaired individuals in their study were significantly less likely to attend a dentist for regular dental checkups than the reference group (37% vs. 54%) [20]. In our study, only about 17% of both the Project participants and the control individuals visited the dentist for regular checkups.

Surprisingly, in our research, oral hygiene habits, including the frequency of toothbrushing, flossing, and the use of fluoridated toothpaste, were not correlated with DMFT. The frequency of dental visits showed correlation with the F-component, suggesting more filled teeth in individuals with more frequent dental visits. The reasons for the last dental visits correlated with the D and M components, suggesting more decayed and extracted teeth in individuals visiting the dentist due to pain caused by teeth/gums or dental treatment—waiting for pain before seeking dental care leads to more severe oral health problems.

A significant correlation was found between decayed teeth (F-component) and soft drink/lemonade consumption (*p* = 0.000) and tobacco use (*p* = 0.014); however, these correlations did not reflect on the total DMFT scores. Maciel et al., in their study of individuals attending the Paraiba Institute of the Blind in Brazil, also found higher DMFT in visually impaired individuals who smoked, but it was under the statistical level of significance [33]. Food and beverages containing sugar are well-known risk factors for caries [37]. Research has also shown that tobacco use is one of the most prevalent public health problems due to its negative influence on both systemic and oral health [38].

Schembri & Fiske investigated oral health in 62 elderly people (aged 60–100, with a mean of 63.5) with visual impairment in Malta, of whom 45% were edentulous [23]. Only 39% brushed teeth daily, while 28% reported brushing weekly. All of them (100%) sought dental treatment only when they experienced problems. In our study sample, among the 34 participants aged 60+, only two individuals were edentulous, indicating better oral healthcare in Croatia in comparison to Malta.

The DMFT level with a median score of 16 in individuals with normal vision in our study is comparable with the results of the Danish national survey from 2007 to 2008, which recorded a mean DMFT value of 18.9 [39]. It is surprising that the Croatian DMFT is lower, since Denmark is a more economically developed country and has not been affected by war in the past 80 years. This is possibly a reflection of different health care systems, with the Croatian system yielding better results. A decrease in the prevalence of caries among adults has been reported in the neighbouring Slovenia [40]. In 30 years, the average DMFT decreased from 17.5 to 15.7 in 45-year-olds, 20.4 to 19.2 in 55-year-olds, and from 22.5 to 20.7 in 65-year-olds. The mean of recent DMFT values of these three age groups is 18.5, while in our control sample’s 45–64 age group it is 20 (Table 4), which is very similar.

As in our research caries experience expressed through DMFT score did not correlate with oral hygiene habits, time since the last dental visit, or level of education, we must seek explanation in other factors. Self-reported oral hygiene information is prone to limitations, including overreporting positive behaviours and underreporting negative ones, and inaccurate recall bias, so this could be a possible explanation. Additionally, frequent toothbrushing but with improper technique, as observed in the Project, may result in inadequate oral hygiene. Another explanation may lie in the organization and specificities of oral healthcare. Croatia has been a European Union member country since 2013. Although it ranks among the high-income countries according to the World Bank, in 2023 Croatia’s GDP per capita reached only 76% of the EU average [41]. Economic development was strongly affected by the Croatian War of Independence from 1991 to 1995. In 1991, systematic preventive oral health care was discontinued and has never been reinstated to cover the entire paediatric population. Dental care is covered through public health insurance and is free for children, while for adult citizens, conservative treatment is mostly free, with a minimal participation fee only for aesthetic fillings in posterior teeth and specialist treatments. Free and available dental care explains a low number of decayed and high numbers of filled teeth in our Project participants and the control group. Another possible explanation is the attitude toward dental health, which is often not valued as highly as general health. While having a hole in one’s nose would not be acceptable to most people, developing a cavity in a tooth, and having it filled or even extracted, is perceived as normal [42]. Another possible explanation might be the sample size, which may not have been large enough to reach statistically significant correlations.

Decades of research and practice in preventive methods have demonstrated that the most rational and efficient method for prevention and control of dental caries and periodontal diseases is daily mechanical cleaning of all tooth surfaces by self-care [42,43]; therefore, individual oral hygiene instruction should be an obligatory task of the dental team. As caries is a multifactorial disease to which people remain susceptible throughout their lifetime, continuous care, support, and motivation for oral hygiene and healthy lifestyle are essential. Unfortunately, it appears that few dentists in Croatia dedicate sufficient time to addressing these needs, as the oral healthcare system is primarily oriented toward curative dentistry, with no allocated time for oral hygiene instruction. In some countries, dental hygienists are responsible for such education. In Croatia, this profession has only recently been introduced and is not yet included in the workforce systematization of dental offices and clinics. Another problem is the absence of a recall system and no control over the frequency of dental visits, which are essential, as patients need to be monitored for life-changing factors related to oral diseases and motivated for oral hygiene.

People with disabilities are a vulnerable group requiring special attention and support in healthcare to overcome barriers and preserve both oral and overall health. There are four categories of barriers that prevent access to oral care: individual; dental professional; societal, and governmental barriers [44]. Individual barriers include a lack of perception of oral health needs by individuals or their caregiver’s, difficulty with oral self-care, and access problems. Barriers related to dental professionals include lack of education, poor communication skills, high staff turnover with consequent lack of trust and continuity of care, and a lack of time and finances [44,45]. To overcome individual barriers, it is necessary to: (1) provide oral health education to individuals and their caregivers; (2) train patients in oral hygiene; and (3) ensure annual dental checkups [46,47]. Involving students in preventive recall care can serve as a source of workforce, enabling continuous care for a high volume of special needs patients and reducing oral health inequalities [48,49,50,51]. With this approach, people with disabilities would be empowered to preserve and retain their health and enjoy the improved quality of life it brings. Additionally, the final cost of dental services would be lower.

There is a lack of information regarding dental health in adults with visual impairment; therefore, this study is important as it evaluates both caries experience and oral health-related habits and offers a comparative perspective with a control group matched by age, sex, and education level. It shows gaps in oral health knowledge and their repercussions, so it can serve for planning future interventions to improve both dental health and overall well-being. Another advantage is that this research was part of a preventive Project, with direct benefits of prophylactic and preventive treatment, and individual oral hygiene instructions provided for all participants, including both the study and control groups.

As participation in the Project was on a voluntary basis and participants were self-selected, this may have led to some response bias towards participants who are more active, better educated, and more interested in their oral health. Previous investigation revealed low levels of dental anxiety in the same sample, indicating that individuals with clinically relevant dental anxiety did not enrol in the Project [52].

Another limitation of this study is that the sample included predominantly visually impaired individuals living in the capital city of Zagreb. It is possible that our study participants had better access to healthcare and possibly better oral health than we would find in a random sample of visually impaired people across Croatia.

## 5. Conclusions

In the present research, the DMFT score revealed a high caries experience among both visually impaired and control individuals, which did not correlate with sex, category of visual impairment, self-reported oral hygiene habits, or education. However, the D, M, and F components were correlated with soft drink consumption, dental visits, and smoking. The high caries experience in both groups indicates the necessity for early and continuous preventive dental care. Oral health promotion activities are needed to raise awareness about the importance of oral health and to provide education on oral hygiene, preventive measures, and regular dental visits. This is necessary for the whole population, but especially for people belonging to vulnerable groups.

It is hoped that our study will provide insights and data useful for raising awareness among healthcare providers and policy makers regarding the approach and necessity of preventive dental care for this and other vulnerable populations.

## Figures and Tables

**Table 1:** Sample stratification by visual impairment category and age of onset (number of individuals).

Visual Impairment Category	Age of Onset	Blind	Low Vision
Blindness	At birth	8	0
Progressive visual impairment	At birth	18	11
	3–10 years	5	4
	11–20 years	5	5
	21–30 years	3	2
	31–40 years	3	3
	41–50 years	1	3
	51–65 years	5	2
	66+ years	2	3
No answer		0	2
Total		50	35

**Table 2:** Study participants’ demographic data: number of individuals (N) and percentage distribution (%).

		N	%
Study Group	Blind	50	58.8
	Low vision	35	41.2
Sex	Male Female	43 42	50.6 49.4
Age groups (Years)	18–34	21	24.7
	35–44	9	10.6
	45–64	25	29.4
	65–74	22	25.9
	75+	8	9.4
Place of residence	Urban	70	82.4
	Peri urban	10	11.8
	Rural	5	5.9
Education level	No formal schooling	0	0.0
	Unfinished primary school	1	1.2
	Primary school	9	10.6
	Secondary school	44	51.8
	High school (gymnasium)	5	5.9
	College/University Postgraduate (MS/PhD)	23 3	27.1 3.5

**Table 3:** Caries experience expressed as decayed (D), missing (M), and filled (F) teeth, and the total DMFT score in study and control groups, Mann–Whitney test.

							Percentile			
	Group	N	Mean	SD	Min	Max	25th	50th (Median)	75th	*p*
D	Study	85	1.7	2.0	0.0	12.0	0.0	1.0	3.0	0.85
	Control	85	1.8	2.1	0.0	9.0	0.0	1.0	3.0	
F	Study	85	6.7	5.3	0.0	19.0	2.0	6.0	10.0	0.63
	Control	85	6.1	4.9	0.0	17.0	2.0	5.0	10.0	
M	Study	85	8.6	8.1	0.0	32.0	2.0	7.0	14.0	0.69
	Control	85	8.4	8.5	0.0	32.0	1.0	7.0	11.5	
DMFT	Study	85	17.1	7.0	1.0	32.0	12.5	17.0	22.0	0.55
	Control	85	16.4	6.9	0.0	32.0	12.0	16.0	21.0	

Mann–Whitney test.

**Table 4:** Study group DMFT values by categories of visual impairment, sex, age, place of residence, and education (^1^ Mann–Whitney test, ^2^ Kruskal–Wallis test).

		D	F	M	DMFT
		Median (IQR)	Median (IQR)	Median (IQR)	Median (IQR)
Study Group ^1^	Blind (n = 50)	1.0 (0.0–3.0)	6.0 (1.7–11.0)	6.5 (2.0–14.0)	17.0 (12.7–22.2)
	Low vision (n = 35)	1.0 (0.0–2.0)	7.0 (2.0–10.0)	7.0 (1.0–15.0)	17.0 (12.0–22.0)
*p*		0.621	0.754	0.890	0.947
Sex ^1^	Male (n = 43)	1.0 (0.0–3.0)	7.0 (2.0–11.0)	7.0 (3.0–15.0)	18.0 (14.0–23.0)
	Female (n = 42)	1.0 (0.0–3.0)	6.0 (1.0–9.2)	5.5 (1.0–12.2)	16.0 (11.0–20.0)
*p*		0.595	0.402	0.354	0.177
Age Groups ^2^ (yrs)	18–34 (n = 21)	2.0 (0.0–4.5)	7.0 (2.5–8.5)	0.0 (0.0–2.0)	11.0 (5.0–14.5)
	35–44 (n = 9)	1.0 (1.0–2.5)	7.0 (4.0–13.0)	4.0 (1.0–7.5)	16.0 (11.5–18.5)
	45–64 (n = 25)	2.0 (0.0–3.0)	10.0 (2.0–13.0)	9.0 (3.0–11.0)	20.0 (15.0–23.0)
	65–74 (n = 22)	1.0 (0.0–2.2)	4.5 (0.0–9.2)	14.5 (6.7–16.2)	17.5 (15.5–22.5)
	75+ (n = 8)	0.0 (0.0–0.7)	2.5 (0.0–9.7)	22.0 (10.5–25.7)	25.0 (22.5–29.7)
*p*		0.151	0.076	<0.001	<0.001
Place of residence ^2^	Urban (n = 70)	1.0 (0.0–2.2)	6.5 (2.0–11.0)	6.0 (2.0–13.0)	17.0 (12.7–20.0)
	Periurban (n = 10)	2.0 (0.7–3.5)	6.0 (1.5–8.2)	10.0 (3.0–20.2)	23.0 (11.7–25.0)
	Rural (n = 5)	2.0 (0.5–5.5)	10.0 (0.0–10.5)	9.0 (4.5–18.0)	19.0 (14.5–25.5)
*p*		0.192	0.753	0.407	0.337
Education level ^2^	Primary school (n = 9)	2.0 (0.5–5.0)	0.0 (0.0–8.0)	15.0 (1.5–23.5)	17.0 (14.5–25.5)
	Secondary school (n = 44)	1.0 (0.0–2.7)	6.0 (2.0–10.7)	9.0 (5.0–13.0)	18.0 (14.2–22.0)
	High school/Gymnasium (n = 5)	0.0 (0.0–2.5)	7.0 (3.5–8.0)	0.0 (0.0–2.0)	8.0 (4.00–11.50)
	College/University (n = 23)	2.0 (0.0–3.0)	8.0 (1.0–13.0)	3.0 (0.0–14.0)	16.0 (11.0–23.0)
	Postgraduate (MS/PhD) (n = 3)	2.0 (0.0–3.0)	7.0 (6.0–14.0)	3.0 (2.0–10.0)	17.0 (11.0–19.0)
*p*		0.589	0.303	0.078	0.069

^1^ Mann–Whitney test, ^2^ Kruskal–Wallis test.

**Table 5:** Experience of toothache; frequency and reasons for dental visits; oral hygiene habits. (Fisher–Freeman exact test).

		Study Group		Control Group		
		N	%	N	%	*p*
Toothache or discomfort during past 12 months	No answers	0	0.0%	0	0.0%	0.323
	Yes (often, occasionally, rarely)	49	57.6%	58	68.2%	
	Never	34	40.0%	25	29.4%	
	Do not know	2	2.4%	2	2.4%	
Frequency of toothbrushing	Never	1	1.2%	0	0.0%	0.505
	Once a month	0	0.0%	0	0.0%	
	2–3 times a month	0	0.0%	0	0.0%	
	Once a week	1	1.2%	0	0.0%	
	2–6 times a week	3	3.5%	1	1.2%	
	Once a day	18	21.2%	22	25.9%	
	2 or more times a day	62	72.9%	62	72.9%	
Use of fluoridated toothpaste	Yes	48	56.5%	22	25.9%	<0.001
	No	5	5.9%	7	8.2%	
	Do not know	32	37.6%	56	65.9%	
Use of dental floss	Yes	24	28.2%	33	38.8%	0.193
	No	61	71.8%	52	61.2%	
Last visit to the dentist	<6 months	35	41.2%	42	49.4%	0.157
	6–12 months	18	21.2%	19	22.4%	
	1–2 years	17	20.0%	9	10.6%	
	2–5 years	7	8.2%	12	14.1%	
	>5 years	8	9.4%	3	3.5%	
	No visit	0	0.0%	0	0.0%	
Reason of the last visit to the dentist	Consultation/advise	6	7.1%	5	5.9%	0.798
	Pain/troubles with teeth or gums	32	37.6%	27	31.8%	
	Dental treatment or ongoing treatment	32	37.6%	38	44.7%	
	Regular dental visit	15	17.6%	14	16.5%	
	Do not know/Do not remember	0	0.0%	1	1.2%	

**Table 6:** Dietary habits—consumption of sugary foods and drinks (Fisher–Freeman exact test).

		Study Group		Control Group		
		N	%	N	%	*p*
Fresh fruits	Never	1	1.2%	1	1.2%	0.788
	Several times per month	7	8.2%	8	9.4%	
	Once per week	4	4.7%	5	5.9%	
	Several times per week	23	27.1%	30	35.3%	
	Every day	40	47.1%	35	41.2%	
	Several times per day	10	11.8%	6	7.1%	
Biscuits, cakes	Never	5	5.9%	9	10.6%	0.869
	Several times per month	21	24.7%	19	22.4%	
	Once per week	13	15.3%	14	16.5%	
	Several times per week	30	35.3%	26	30.6%	
	Every day	15	17.6%	15	17.6%	
	Several times per day	1	1.2%	2	2.4%	
Pies, buns	Never	12	14.3%	11	12.9%	0.758
	Several times per month	28	33.3%	26	30.6%	
	Once per week	12	14.3%	19	22.4%	
	Several times per week	25	29.8%	22	25.9%	
	Every day	7	8.3%	7	8.2%	
	Several times per day	0	0.0%	0	0.0%	
Jam, honey	Never	14	16.5%	22	25.9%	0.146
	Several times per month	29	34.1%	25	29.4%	
	Once per week	5	5.9%	12	14.1%	
	Several times per week	21	24.7%	15	17.6%	
	Every day	16	18.8%	11	12.9%	
	Several times per day	0	0.0%	0	0.0%	
Chewing gum (containing sugar)	Never	57	67.1%	67	78.8%	0.219
	Several times per month	14	16.5%	8	9.4%	
	Once per week	2	2.4%	4	4.7%	
	Several times per week	8	9.4%	5	5.9%	
	Every day	3	3.5%	0	0.0%	
	Several times per day	1	1.2%	1	1.2%	
Sweets, candies	Never	21	24.7%	20	23.5%	0.616
	Several times per month	30	35.3%	23	27.1%	
	Once per week	5	5.9%	10	11.8%	
	Several times per week	19	22.4%	21	24.7%	
	Every day	10	11.8%	10	11.8%	
	Several times per day	0	0.0%	1	1.2%	
Soft drinks, lemonade	Never	25	29.4%	29	34.1%	0.442
	Several times per month	32	37.6%	24	28.2%	
	Once per week	3	3.5%	8	9.4%	
	Several times per week	19	22.4%	17	20.0%	
	Every day	6	7.1%	6	7.1%	
	Several times per day	0	0.0%	1	1.2%	
Milk with sugar	Never	70	82.4%	70	82.4%	0.138
	Several times per month	8	9.4%	2	2.4%	
	Once per week	1	1.2%	2	2.4%	
	Several times per week	4	4.7%	3	3.5%	
	Every day	2	2.4%	6	7.1%	
	Several times per day	0	0.0%	2	2.4%	
Tea/coffee with sugar	Never	43	50.6%	58	68.2%	0.152
	Several times per month	16	18.8%	9	10.6%	
	Once per week	6	7.1%	2	2.4%	
	Several times per week	11	12.9%	8	9.4%	
	Every day	9	10.6%	8	9.4%	
	Several times per day	0	0.0%	0	0.0%	

**Table 7:** Tobacco and alcohol use (Fisher–Freeman exact test).

		Study Group		Control Group		
		N	%	N	%	*p*
Tobacco use	Never	69	81.2%	61	71.8%	0.554
	Rarely	0	0.0%	1	1.2%	
	Several times per month	1	1.2%	1	1.2%	
	Once per week	0	0.0%	1	1.2%	
	Several times per week	0	0.0%	0	0.0%	
	Every day	15	17.6%	21	24.7%	
Alcohol use per day	<1 drink	4	4.7%	1	1.2%	0.399
	One drink	21	24.7%	27	31.8%	
	Two drinks	15	17.6%	10	11.8%	
	Three drinks	8	9.4%	11	12.9%	
	Four drinks	1	1.2%	4	4.7%	
	>5 drinks	4	4.7%	2	2.4%	
	No alcohol during past month	32	37.6%	30	35.3%	

**Table 8:** Correlation of DMFT scores and their components with oral health-related habits in blind and low-vision participants (Spearman’s correlation test).

		D	F	M	DMFT
Toothache or discomfort during past 12 months	Correlation Coefficient	0.082	−0.105	−0.133	−0.098
	*p*	0.460	0.346	0.231	0.379
Frequency of toothbrushing	Correlation Coefficient	−0.070	−0.082	−0.061	−0.093
	*p*	0.526	0.456	0.577	0.397
Fluoridated toothpaste	Correlation Coefficient	0.056	−0.134	0.198	0.139
	*p*	0.608	0.221	0.070	0.204
Last visit to dentist	Correlation Coefficient	0.003	−0.453	0.114	−0.103
	*p*	0.976	0.000 ***	0.297	0.347
Reason of dental visit	Correlation Coefficient	−0.269	0.207	−0.245	−0.115
	*p*	0.013 *	0.058	0.024 *	0.295
Fresh fruit	Correlation Coefficient	−0.135	−0.049	0.026	−0.027
	*p*	0.218	0.655	0.817	0.807
Biscuits, cakes	Correlation Coefficient	0.024	0.163	0.021	0.041
	*p*	0.830	0.135	0.849	0.711
Pies, buns	Correlation Coefficient	0.183	0.103	0.025	0.033
	*p*	0.095	0.353	0.823	0.766
Jam, honey	Correlation Coefficient	−0.071	−0.062	0.148	0.113
	*p*	0.516	0.571	0.177	0.304
Chewing gum (containing sugar)	Correlation Coefficient	0.045	0.080	−0.205	−0.186
	*p*	0.684	0.469	0.060	0.089
Sweets, candies	Correlation Coefficient	0.113	−0.023	0.165	0.156
	*p*	0.305	0.835	0.132	0.155
Soft drinks, lemonade	Correlation Coefficient	0.374	0.015	−0.110	−0.042
	*p*	0.000 ***	0.890	0.318	0.703
Milk with sugar	Correlation Coefficient	−0.082	−0.009	0.195	0.144
	*p*	0.456	0.938	0.073	0.190
Coffee/Tea with sugar	Correlation Coefficient	−0.036	0.008	0.105	0.096
	*p*	0.745	0.944	0.339	0.383
Smoking	Correlation Coefficient	0.265	−0.031	−0.031	−0.023
	*p*	0.014 *	0.779	0.780	0.837
Alcohol	Correlation Coefficient	0.015	−0.124	−0.037	−0.018
	*p*	0.892	0.258	0.734	0.870

Statistically significant *p*-values marked bold; * *p* < 0.05, *** *p* < 0.001.

## Data Availability

The original contributions presented in this study are included in the article/Supplementary Material. Further inquiries can be directed to the corresponding author(s).

## Supplementary Materials

The following supporting information can be downloaded at https://www.mdpi.com/article/10.3390/jcm14155576/s1, Research dataset Caries Experience and Oral Health-Related Habits in Blind and Low-Vision Individuals in Croatia. Table S1: Study group and case controls dental status and WHO Oral Health Questionnaire results [32]. Coding: 0 Sound, 1 Caries, 2 Filled, with caries, 3 Filled, no caries, 4 Missing due to caries, 5 Missing for any other reason, 6 Fissure sealant, 7 Fixed dental prosthesis abutment, special crown or veneer/implant, 8 Unerupted tooth (crown)/unexposed root, 9 Not recorded. Tooth notation according to FDI: Z18 to Z48. WHO Oral Health Questionnaire responses: Q1–Q16.

## Abbreviations

The following abbreviations are used in this manuscript:

D	Number of decayed teeth
DMFT	Decayed, Missing, and Filled Teeth
F	Number of filled teeth
ICC	Intraclass Correlation Coefficient
M	Number of missing teeth
UK	United Kingdom
WHO	World Health Organization
